# Vulvar Leiomyosarcoma in Pregnancy

**DOI:** 10.7759/cureus.18772

**Published:** 2021-10-14

**Authors:** Ala M Aljehani, Amani Quatei, Lina Qattea, Renad M Aljohani, Abdulmohsen Alkushi

**Affiliations:** 1 Medicine, Imam Mohammad Ibn Saud Islamic University, Riyadh, SAU; 2 Obstetrics and Gynecology, King Fahad Medical City, Riyadh, SAU; 3 Obstetrics and Gynecology, King Abdulaziz Medical City Riyadh, Riyadh, SAU; 4 Medicine, King Saud bin Abdulaziz University for Health Sciences, Riyadh, SAU; 5 Pathology and Laboratory Medicine, King Saud bin Abdulaziz University for Health Sciences, Riyadh, SAU; 6 Pathology and Laboratory Medicine, King Abdulaziz Medical City, Riyadh, SAU

**Keywords:** leiomyosarcoma, vulva, pregnancy, bartholin's cyst, malignancy

## Abstract

Vulvar leiomyosarcoma is a rare smooth muscle malignant neoplasm but it is the commonest type of vulvar sarcomas. It may mimic benign tumors and misdiagnosis could delay proper management. We report a case of a 38-year-old pregnant woman with leiomyosarcoma of the vulva. The patient presented to her primary general practitioner with a small vulvar mass that she had first noticed one year prior. The tumor was suspected to be benign Bartholin’s cyst and treated with antibiotics. The patient declined improvement and had many consultations to different clinics where she had been diagnosed and treated the same. The tumor size started to grow rapidly after she got pregnant, and the patient was referred to our hospital where she underwent tumor resection. Histopathology revealed leiomyosarcoma. The patient had further assessment and close follow-up and has had no recurrence for 12 months. There is little literature available on vulvar leiomyosarcoma, most of which are case reports, and most gynecologic oncologists will go through their whole careers without seeing a single case.

## Introduction

Vulvar sarcomas are rare representing 1-3% of all malignancies arising in the vulva. They are of a mesenchymal origin and the most common types of vulvar sarcomas are leiomyosarcoma, rhabdomyosarcoma, liposarcoma, angiosarcoma, malignant peripheral nerve sheath tumor, malignant fibrous histiocytoma, and epithelioid sarcoma [1-4].

Leiomyosarcoma is the most common sarcoma of the vulva. It is a rare malignant neoplasm of the smooth muscle. Furthermore, its association with pregnancy is very rare. Limited data is available about its biological behavior, prognosis, and treatment strategies in pregnancy.

Especially given the increasing incidence over the past several decades, however, this increase may be attributed to genetic predisposition, lifestyle, and sexual habits [2, 3]

In an attempt to add to the scarce literature data on this rare tumor, we are presenting this case report. A review of the course, incidence, diagnosis, and therapeutic approach of leiomyosarcoma of the vulva in pregnancy will be provided in this case report.

## Case presentation

A 38-year-old woman, gravida 5 para 4 presented at 32 weeks pregnancy. She first presented to her primary general practitioner with a small vulvar lesion that she had first noticed one year prior. The lesion was suspected to be benign Bartholin’s cyst and treated with broad-spectrum antibiotics. The patient noticed no improvement and had many other consultations with different clinics when she was advised to the same diagnosis and treatment. Initially, the swelling was constant in size, however, it started to enlarge rapidly. A significant increase in size was noticed during pregnancy and causing a restless feeling. The patient was thus referred to our hospital. She had no significant gynecological past history, prior gynecological malignancies, or condyloma acuminatum. Her pregnancy course was uneventful. The medical, surgical, and family histories were noncontributory. No medications had been taken before or during the pregnancy.

**Figure 1:**
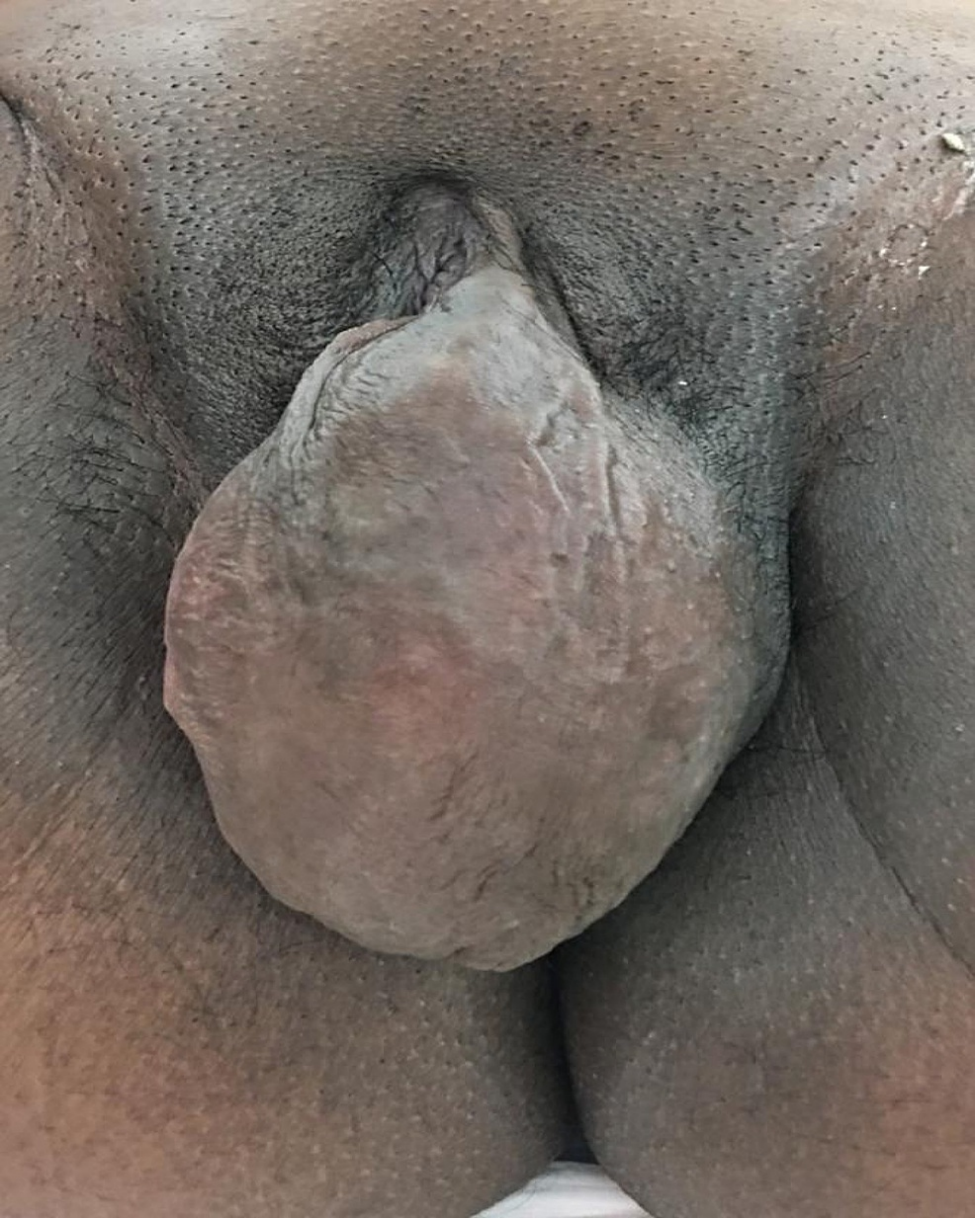
The mass before operation

Physical examination revealed a healthy woman with normal vital signs. Abdominal examination reveals a gravid abdomen with a fetus of appropriate size for the gestational age in cephalic presentation. Pelvic examination revealed a vulvar mass at the left side involving left labia majora and minora. The mass was painless, rounded, firm in consistency, freely mobile with an intact overlying skin measuring 15.0 x 10.0 cm diameter (Figure 1).

No palpable lymph node was detected. Pelvic Magnetic Resonance Imaging (MRI) with heterogeneous signal intensity on T2-weighted images showed a solid mass with no cystic signal intensity measuring 4.8 x 7.2 x 10 cm. The radiological impression was labial leiomyoma. 

**Figure 2:**
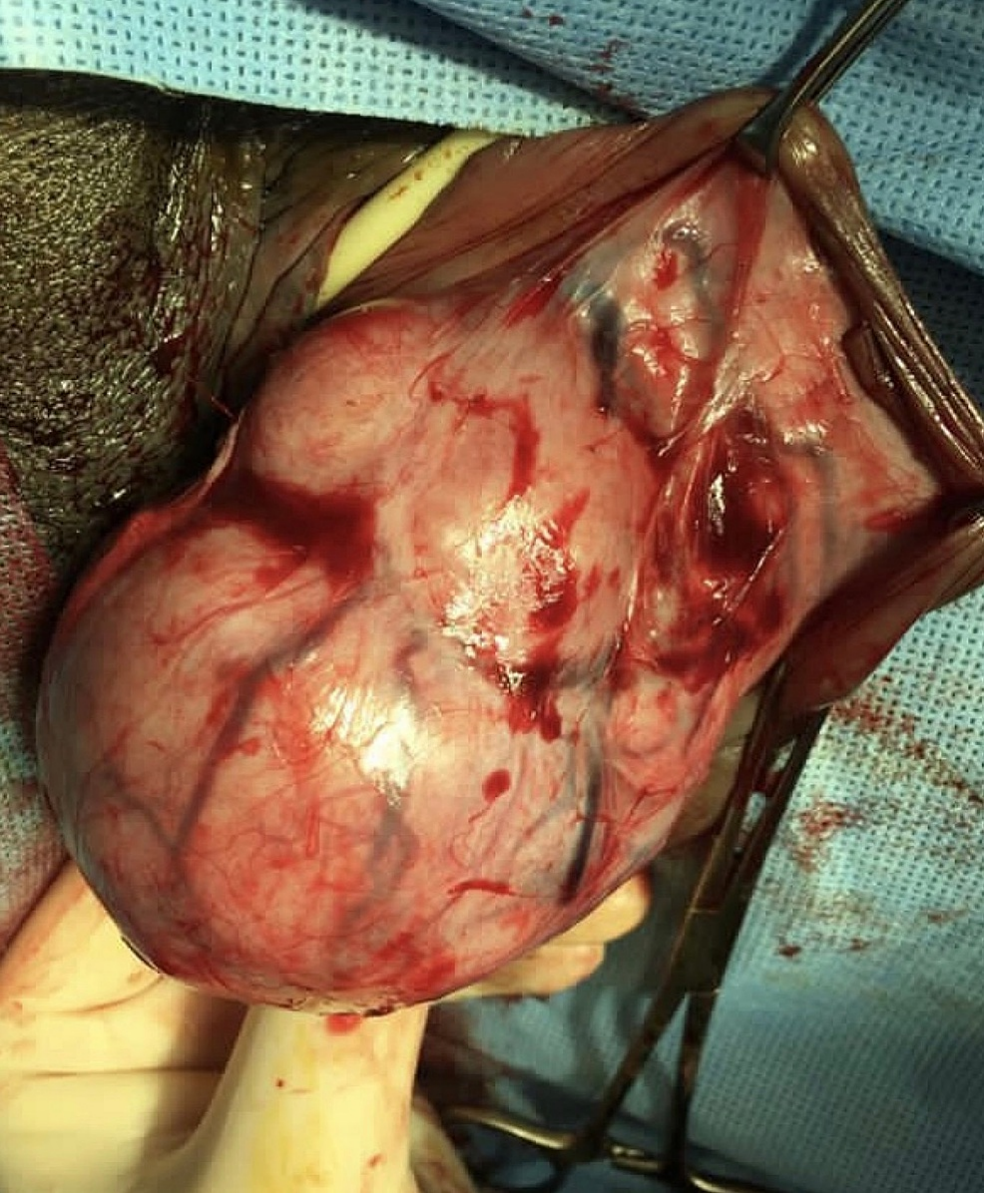
The mass during operation

**Figure 3:**
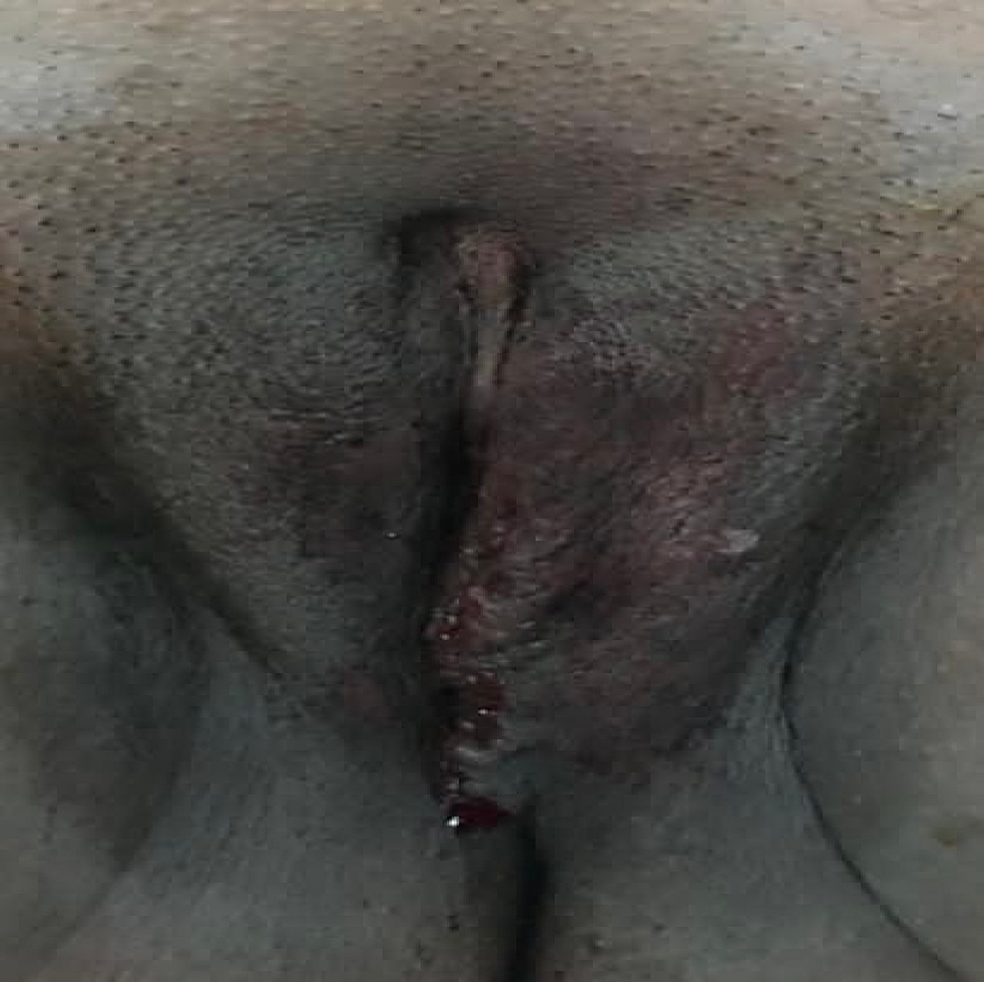
After operation

At 34 weeks of pregnancy, the patient was admitted for tumor resection. A baseline fetal cardiotocography and obstetrics ultrasound were normal. Under spinal analgesia, the tumor was completely resected with minimal blood loss (Figures 2, 3). No frozen section samples were taken. The patient and her fetus tolerated the procedure well. The histopathology report revealed leiomyosarcoma. The cease was discussed with the gynae-oncology team and the patient as well and a plan was agreed for induction of labor for further radiological assessment and treatment. Therefore, the patient was induced at 34 weeks and had a vaginal delivery; the antepartum and postpartum course were unremarkable.

The patient was referred after delivery to the Gynaeoncology department for follow-up and further assessment and management. She had pelvic MRI, abdominopelvic CT with contrast, PET-CT head, neck, and lower extremity and show no tumor deposit, neither local nor distal. On post-surgical follow-up, the patient was asymptomatic and had no radiotherapy or adjuvant chemotherapy. She had no evidence of recurrence or distant metastasis on 12 months follow-up after surgery.

Histopathology

**Figure 4:**
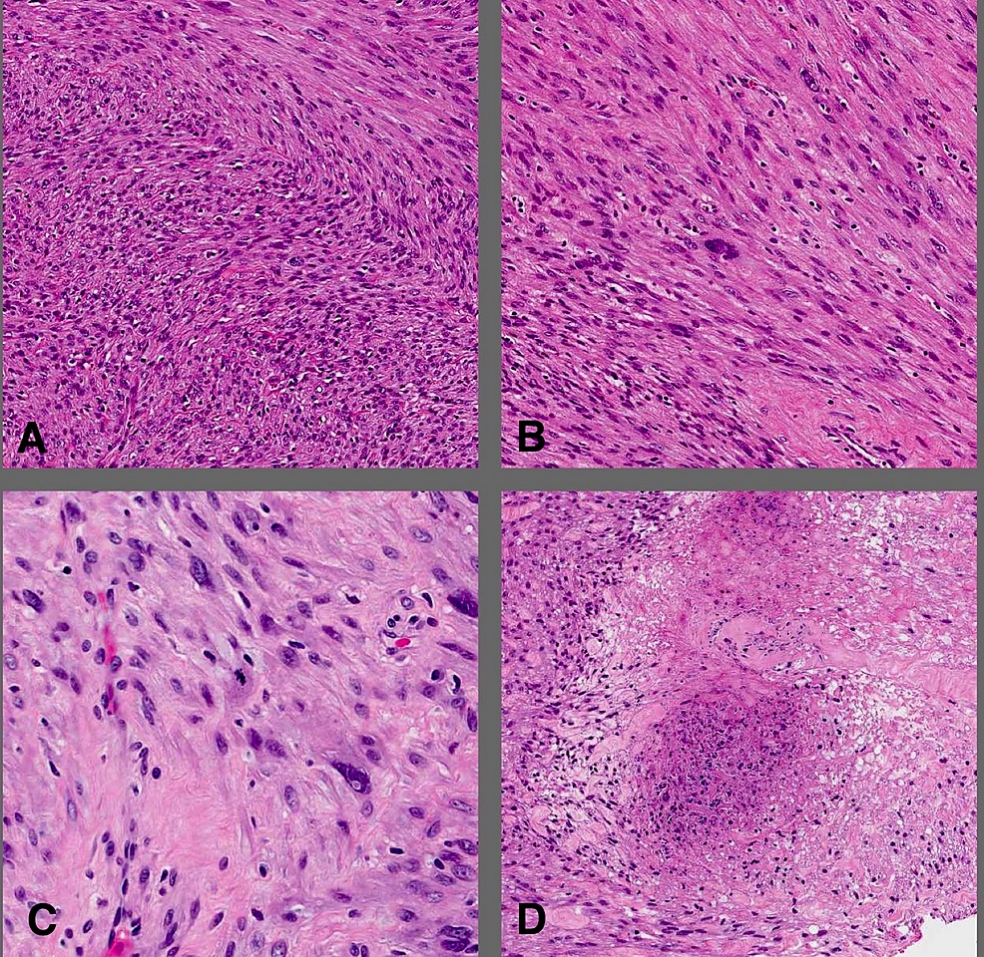
H&E stained sections from the mass Neoplastic cells are arranged in intersecting fascicles (A) (10x). Nuclear atypia, mitosis, and necrosis are shown in (B, C, and D), respectively (B, D - 20x) (C - 40x).

**Figure 5:**
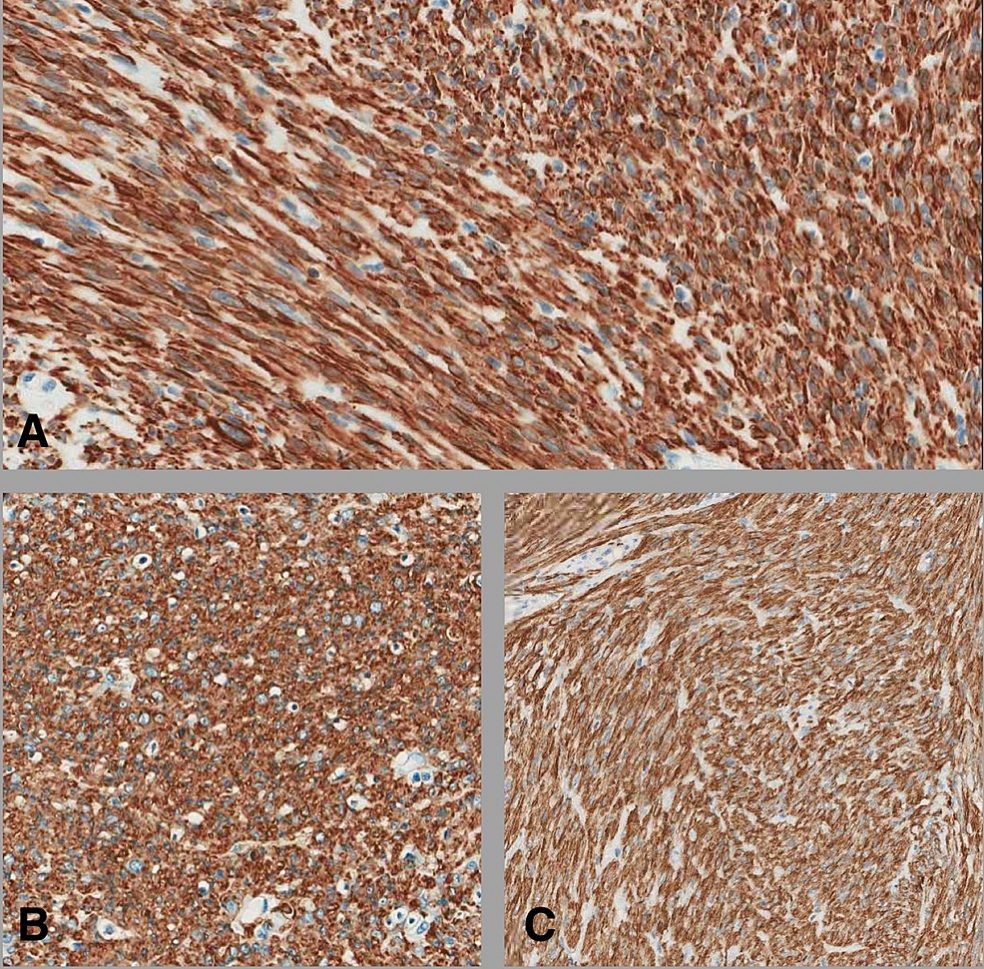
Immunostains visible for Desmin (A), SMA (B) and Caldesmin (C) (20x) SMA: smooth muscle actin

Gross evaluation revealed a well-defined mass measuring 10.0 x 8.0 x 5.0 cm and showing tan whorly cut surfaces with no obvious areas of hemorrhage or necrosis. Histological examination showed smooth muscle neoplasm arranged in fascicles. The tumor cells showed mild to moderate nuclear atypia and increased cellularity. The mitotic index was nine mitotic figures per 10 high power fields (HPF) when the high method is used. Small foci of tumor necrosis were identified (Figure 4). The tumor cells were positive for smooth muscle markers (smooth muscle actin [SMA], Desmin, and Caldesmon) (Figure 5). Based on the above-mentioned findings and applying the current WHO classification the case was diagnosed as leiomyosarcoma.

## Discussion

This case is the first case of vulvar leiomyosarcoma showing a rapid increase in size during pregnancy reported in the Gulf region. The literature review revealed few documented cases of leiomyosarcoma located in the female genital tract [1-7].

The deleterious effect of pregnancy on the course of leiomyosarcoma remains a matter of dispute among the authors. Several reports contain evidence that pregnancy may have a deleterious effect on the course of the tumor, however, this is not well supported through adequate data.

The presentation can vary according to the stage of disease, but generally, patients will present predominantly with an asymptomatic, slowly growing vulvar mass, pain, discomfort, bleeding, and voiding difficulty with a size range between 1.5-16 cm [3,5,7,8]. Women often have difficulty articulating vulvar symptoms to medical practitioners, therefore, all women with vulvar symptoms should be examined. It arises most frequently from the labia majora, followed by, in decreasing order, the Bartholin’s gland area, clitoris, and labia minora with clinical similarities between them leading to difficulties in clinical diagnosis [2,4,5,7,9,10]. The mean age of incidence has been reported to be between 33 and 40 years, with a range between 15 and 84 years [3]. It can recur both locally and distantly over a long period. Our patient presented with a painless, initially slow-growing swelling in the left labia majora then rapidly increased in size during pregnancy over one year. The biological behavior of leiomyosarcoma in the vulva is identical to that in other locations. In general, the disease may follow a short and aggressive or a protracted course with late recurrences [9,11].

Many vulvar cancers are initially misdiagnosed as inflammatory conditions, Bartholin’s gland cyst, lipoma, fibroma, or leiomyoma, which could delay the diagnosis and worsening prognosis [2,4,5,7]. Diagnostic and staging workup should be carefully performed for appropriate maternal and fetal management. Recommendation of imaging studies should always follow the established guidelines for pregnant patients.

The final diagnosis of leiomyosarcoma is usually done on histopathological examination of the tumor. Pregnancy may induce histologic changes that may confuse the examining pathologist, therefore notifying the pathologist with pregnancy status is essential as it may require careful assessment.

There are two classification systems that are used for smooth muscle tumor (SMT) of the vulva (site-specific criteria). In 1979, Tavassoli and Norris issued the first criteria for vulvar SMTs [12], and in 1996, Nielsen and others issued another criterion [9]. However, most histopathologists tend to implement the criteria for uterine SMTs when investigating a vulvar tumor [13]. According to the 2020 World Health Organization (WHO) classification of Tumors of Female Reproductive Organs [13], three main elements are evaluated when determining the type of SMT of the uterine: mitotic index, tumor cell necrosis, and the extent of cytological atypia. Leiomyosarcoma is diagnosed when no less than two of the three elements are recognized: mitotic index ≥ 10 per 10 HPFs, moderate to severe cytological atypia, and tumor cell necrosis [14]. Recently Sayeed and colleges [15] found that uterine criteria were as sensitive as and more specific than site-specific criteria. And the current WHO classification applies the same criteria used for uterine SMT [13].

Surgery is the cornerstone of therapy for vulvar cancer, but because of its rarity, evidence-based treatment algorithms are not available [1-3]. The treatment of choice for leiomyosarcomas is complete wide local excision with a goal of pathologic confirmation of negative margins 1-2 cm normal tissue this will prevent regional and distal metastasis which make the prognosis worse if happened [5,7,11]. Prophylactic lymph node dissection in operable cases is not recommended. The latter is considered overtreatment for leiomyosarcoma of the vulva [10]. Additional measures may include hemivulvectomy, ipsilateral inguinal lymphadenectomy radiation, or chemotherapy at the discretion of the oncologist. The necessity of adjuvant therapy is unknown currently [5,7,9,16]. The local recurrence and frequency of metastasis by the hematogenous route are high with a very aggressive nature and poor prognosis, However, the course of the disease and late recurrence are still not clear [11]. The risk of local recurrence did not relate to the size of the tumor but to inadequate resection margins. Metastatic transmission to the products of conception happens rarely [11]. Our patient underwent wide local tumor resection without lymph node dissection based on the clinical diagnosis and MRI assessment of vulvar leiomyoma with the histopathology report of leiomyosarcoma with negative margins. The patient has been under close follow-up with the oncology team for periodic evaluation to detect any local recurrence.

The decision for preterm delivery was based on several factors including the balance between the risk of cancer progression and the risk of delivery of a premature fetus. Leiomyosarcomas are aggressive tumors and patients whose disease appears to be initially confined to the vulva may experience a clinically aggressive disease course [17]. The understanding of leiomyosarcomas in pregnancy is flimsy and not deep enough. Based on the uncertain pathologic nature status and the appearance of a mass clinically there was a concern for residual disease. The patient was extensively counseled concerning the natural history of leiomyosarcomas and the risks of premature delivery at 34 weeks.

## Conclusions

In conclusion, we report a rare case of vulvar leiomyosarcoma diagnosed in young pregnant women with a good outcome for both the mother and the baby. Leiomyosarcoma should be included in the differential diagnosis of vulvar masses. Progressively enlarging vulvar lesions should be biopsied even during pregnancy. Complete excision with pathologic confirmation of negative margins should be the goal. Dissection of unsuspicious lymph nodes is not recommended. Despite its rarity, both gynecologists and pathologists should be aware of vulvar leiomyosarcoma.
